# Change in activity in Baltic clam (*Macoma balthica*) exposed to elevated sulphate concentrations

**DOI:** 10.1007/s10646-025-02977-7

**Published:** 2025-10-14

**Authors:** Xiaoxuan Hu, Christian Ritz, Hansika Sarathchandra, Jouni Taskinen, Juha Karjalainen

**Affiliations:** 1 Department of Biological and Environmental Science, University of Jyväskylä, Jyväskylä, P.O. Box 35 FI-40014 Finland; 2 National Institute of Public Health, University of Southern Denmark, Studiestraede 6, Copenhagen, DK-1455 Denmark

**Keywords:** Sulphate, Effective concentration, Biotest, Bivalve, Behavior

## Abstract

Behavioral endpoints have been increasingly used in ecotoxicology for their relevance to individual fitness and ecological effects. However, due to the lack of standard experimental protocols, behavioral endpoints are mostly excluded when deriving environmental quality guidelines. To increase reliability and comparability, behavioral endpoints need to be developed alongside standard endpoints in experiments incorporating elements of standard protocols. In this study, we explored a low-cost methodology to examine the use of activity as a chronic endpoint in a bivalve species in sulphate toxicity tests, following principles of the standard protocol. Adults and juveniles of Baltic clam (*Macoma balthica*) were exposed to sodium sulphate separately. The adult bivalves were individually inspected for their activity at frequent intervals. Activity was at first inhibited, but the extent of activity inhibition decreased and activity EC10 (10% effective concentration) increased as the exposure continued. In *M. balthica* juveniles, LC10 based on survival was 9001 mg/L sulphate. In *M. balthica* adults, activity EC10s (1713 − 3176 mg/L) were consistently lower than wet mass EC10 (5511 mg/L). Siphonal autotomy in *M. balthica* was observed at similar sulphate levels as the activity-based chronic EC10s. We found that the sensitivity of behavioral endpoints in major ion exposures depended on the duration of exposure and, consequently, they need to be evaluated in a case-by-case scenario in the regulatory ecotoxicological context.

**Supplementary Information:**

The online version contains supplementary material available at 10.1007/s10646-025-02977-7.

## Introduction

Behavioral responses have been increasingly used in ecotoxicology in addition to traditional endpoints such as survival, development and reproduction (Melvin and Wilson 2013; Saaristo et al. 2018). In the aquatic realm, activity patterns of fish, crustaceans, insects, flatworms and bivalves, among other examples, have been assessed as sensitive non-conventional indicators of individual fitness which can be linked to population dynamics or ecological functions (Brodin et al. 2014; Legradi et al. 2018; Simão et al. 2021; Fogliano et al. 2023; Porras-Rivera et al. 2024; Rasmussen et al. 2024). Even small-scale locomotion irregularities in animals can have cascading ecological effects across trophic levels within the context of predator-prey interactions (Langer-Jaesrich et al. 2010; Brodin et al. 2014; Faimali et al. 2017; Saaristo et al. 2018). For example, contaminant-affected fish can become less adept at capturing prey, leading to higher prey abundance and larger prey size, which can cause cascading effects across trophic levels (Saaristo et al. 2018).

Despite the utility and relevance of behavioral endpoints, being comparatively faster and more sensitive than developmental and reproductive endpoints (Amiard-Triquet 2009; Melvin and Wilson 2013), regulatory ecotoxicology has rarely included behavioral studies in chemical risk assessments for their “low relevance” in the regulatory context (Ågerstrand et al. 2020; Ford et al. 2021). Behavioral studies often deal with the absence of standardized guidance documents, leading to the inevitable result of producing non-standard behavioral endpoints that may not be comparable across laboratories (Ford et al. 2021). For a behavioral study to be accepted for regulatory risk assessment, the general advice has been to incorporate elements of standardized studies in test designs and to bring forward the relevant population-level effects (Amiard-Triquet 2009; Ågerstrand et al. 2020; Ford et al. 2021).

Bivalves are often used as biomarkers for their sensitive behavioral changes under stress (Newton and Cope  2006; Hazelton et al. 2013; Hartmann et al. 2016; Vereycken and Aldridge^2023^; Fogliano et al. 2023). Stressed bivalves often engage in avoidance behaviors by closing their valves (Ortmann and Grieshaber 2003; Soucek 2007; Tuttle-Raycraft et al. 2017; Castro et al. 2018). To capture bivalve activity changes, new technological development has allowed direct analysis of valve movement, such as by attaching sensors directly to the shells of animals or using video recordings (Robson et al. 2009; Hartmann et al. 2016; Miller 2022; Belamy et al. 2023). In a much easier and low-cost manner, foot movement under the microscopic observation for five minutes has been used as a standardized acute endpoint in experiments using juvenile freshwater mussels (Hazelton et al. 2013; Wang et al. 2016, 2020; ASTM 2022). Inhibited foot, siphon or valve movement in individual bivalves would have population-level consequences, as it causes restricted feeding, oxygen intake and metabolism, leading to reduced growth and depleted energy reserves in the long term (Ortmann and Grieshaber 2003; Schartum et al. 2017).

Following general guidance of the ASTM E2455–22 document (ASTM 2022) with justified modifications, we exposed adults of the brackish water Baltic clam *Macoma balthica* to elevated major ions (sulphate SO_4_^2−^) in a setting where the test organisms were inspected for five minutes at frequent intervals for activity over four weeks. Major ions are often considered as benign but high levels can lead to sublethal or lethal effects due to overall induced salinity increases or unbalanced ion compositions in the aquatic media (Mount et al. 1997; Goodfellow et al. 2000; Kunz et al. 2013; Karjalainen et al. 2021; Karjalainen et al. 2023). Research investigating how aquatic life reacts to alterations in major ion concentrations is much needed, as various anthropogenic activities have led to elevated levels of major ions in aquatic environments (Cañedo-Argüelles et al. 2019). Anthropogenic sulphate loads into the Baltic Sea have also been significant (Ekholm et al. 2020), whereas the ecological and physiological effects of sulphate on Baltic Sea species have rarely been examined. Baltic Sea fish species, such as the European whitefish, can be vulnerable to anthropogenic sulphate increases and more studies are needed to understand sulphate sensitivity of brackish water species (Mäkinen et al. 2024).

In our study, sulphate exposure experiments were conducted as a case study to see how elevations in major ion concentrations affected activity and growth of a brackish-water bivalve species, *M. balthica*. Toxicity of sulphate has been investigated in the larval stage of freshwater mussels (glochidia) as well as early juvenile stages, with acute studies reporting depressed foot movement and mortality and chronic studies reporting inhibited growth (Gillis 2011; Wang et al. 2016; Wang et al. 2018; Bringolf et al. 2022; Belamy et al. 2023; Hu et al. 2024). Given that many technologically advanced methodologies following bivalve activities may not apply to all laboratories with smaller budgets, we set out to examine if such a more user-friendly method as observations during frequent intervals would yield important information regarding bivalve activities under chronic stress. Bivalve activities were defined as valve movement, such as gaping, or foot movement, such as foot elongation within five minutes of observation (ASTM 2022). We aimed to answer two questions: (1) How does *M. balthica* activity change with time in exposure to elevated salt concentrations? (2) Is *M. balthica* activity sensitive as a chronic endpoint compared to mass-based endpoints?

## Materials and methods

### Study species and population

#### Macoma balthica

Baltic clams (*Macoma balthica*) were collected in late May 2023 from Tvärminne Archipelago, Finland. Sediments were taken from the − 12 m sea bottom with a veen grab and sieved on 1 mm filter to obtain *M. balthica* individuals. The *M. balthica* clams were transported to University of Jyväskylä in 7–8 °C local Baltic Sea water. In the lab maintained at 10 °C, around 200 adult *M. balthica* clams (10–15 mm in length) were placed into 20 L well aerated aquaria with three-centimeter-thick sediments obtained from their original habitats. Three times a week aquaria water was changed in half with local Baltic Sea water and enhanced with algal mix (30 µL/L Shellfish Diet 3500) as feed additional to the sediments. Oxygen (11 mg/L, over 100% saturation from constant aeration, PreSens 4 Micron oxygen meter) and temperature (9.4–9.8 °C) were frequently checked to be at normal levels during the maintenance. Oxygen measurement was corrected using the salinity compensation factor. Before the experiment in September, stock sea water for water change ran out and water change during maintenance was instead conducted using aerated artificial sea water, which was prepared from Instant Ocean Sea Salt mix to reach a similar degree of salinity as the original brackish sea water (5 g/kg). The experiments were conducted in original Baltic Sea water, and before the experiments, *M. balthica* clams were acclimated in separate beakers with 2 L aerated control test water with algal mix for two to three days.

## Chronic exposure

### Test water and test substance

Local water was used to increase the relevance of the study to local conditions. Original Baltic Sea water collected from Tvärminne Archipelago was filtered (Millipore Pellicon and Durapore GVPP 0.22 cassette, pore size 0.22 μm, 1110 mg/L CaCO_3_ hardness) and spiked with sodium sulphate powder (Na_2_SO_4_, Merck, purity ≥ 99.0%) to prepare different concentrations.

Sodium sulphate was used because sodium Na contributes less to the overall salt toxicity than other cations such as potassium K and magnesium Mg (Mount et al. 2016). In terms of ion toxicity, both cations (such as sodium) and anions (such as sulphate) contribute to osmolarity-related toxicity (Erickson et al. 2022). However, the toxicity of sodium salts is primarily affected by the anions (Mount et al. 2016; Erickson et al. 2022). Thus, in our study, toxicity was expressed in terms of the sulphate anion, as is done in typical sulphate toxicity studies (e.g. Wang et al. 2018, 2020; Karjalainen et al. 2023; Hu et al. 2024).

In the preliminary test carried out in July 2023, *M. balthica* juveniles (length < 4 mm) were exposed individually in a 28-day experiment in 6-well microplates following the same experimental setup of Hu et al. (2024), with 12 replicates per concentration. A total of 10 nominal concentrations were prepared: control (500 mg/L), 3000, 6000, 8000, 10,000, 115,000, 13,000, 145,000, 16,000 and 17,500 mg/L sulphate. Water (10 mL per individual) was changed three times a week on the same weekdays with added algal mix Shellfish Diet 3500 (30 µL/L). Solutions for water change were stored in the experimental room and maintained at experimental temperature levels. Oxygen (> 10 mg/L, > 94% saturation after correcting for the salinity compensation factor) and temperature (9.4 ± 0.1 °C) were checked three times a week. At the start, middle and end of the experiment, pH (7.6–8.2) and conductivity were monitored (Supplementary Information Table S1). Activity and mortality at the end of the experiment were documented but not during the experiment.

A total of 9 nominal concentrations were prepared in the definitive test: control (500 mg/L), 1000, 2000, 3000, 4000, 5000, 6000, 8000 and 12,000 mg/L sulphate. At the start, middle and end of each chronic experiment, water samples were analyzed for actual sulphate concentrations in the *M. balthica* experiment (SFS-EN ISO 10304-1:2009, KVVY, Finland). Measured concentrations were within ± 10% deviations from the nominal concentrations and averaged sulphate concentrations across the samples were used as final concentrations in statistical analysis (Supplementary Information Table S1). Water chemistry analysis results for the base test water in the test were obtained (Supplementary Information Table S2).

## Na_2_SO_4_ exposure on Macoma balthica

*M. balthica* activity was defined as moving shells or foot under 5 min. All clams used were actively moving their foot or siphon before the initiation of tests. In addition, *M. balthica* with siphons which retracted as a response to gentle disturbances were also considered as active. *M. balthica* which closed the shells completely with no signs of siphon protrusion were considered as inactive. *M. balthica* which stayed open, motionless and did not react to gentle disturbances by closing, or those which showed decomposing tissues, were considered as dead.

Adult *M. balthica* clams were exposed individually in a 28-day experiment in beakers with 125 mL test solutions in September 2023. The clams were grouped according to size already during maintenance before the experiment, and 72 similarly sized clams were randomly assigned to each of the nine sulphate concentrations, resulting in eight replicates per concentration. The placement of the beakers was randomized in the test room and the experiment was conducted in the dark. All water was changed three times a week on the same weekdays with added algal mix Shellfish Diet 3500 (30 µL/L). Oxygen (> 10 mg/L, > 94% saturation after correcting for the salinity compensation factor) and temperature (9.2 ± 0.2 °C) were checked three times a week, and pH (7.6–7.9) and conductivity (Supplementary Information Table S1) were measured. No sediments were placed into the test beakers. Experiments on *M. balthica* without sediments were recommended in short-term metal exposures (Griscom and Fisher 2002). However, given that *M. balthica* are facultative feeders which can opportunistically switch from deposit-feeding to filter-feeding (Peterson and Skilleter 1994; de Goeij and Luttikhuizen 1998), a chronic exposure without sediments was conducted on *M. balthica* to facilitate observations of foot, siphon or valve movement.

*M. balthica* clams were observed once a week on the same weekday. Observations were made at the same time of the day (starting at ten o’clock in the morning). Dead clams were discarded. Siphon autotomy was recorded when siphons broke off from the clams. An extra group of 23 clams were sampled before the experiment, and their wet mass, dry mass and lengths were measured. After the experiment, clams which were alive were measured for lengths, then dissected and weighed for wet soft tissue mass, dry soft tissue mass and shell mass. Right after each clam was removed from exposure solutions, their shells were patted dry for 2 s on paper towels and wet animal tissues were immediately removed from the shells. Wet tissues were placed on pre-weighed aluminum foil boats for mass measurement. Weighing of wet tissues was conducted at a fast and steady rhythm for all clams to minimize inaccuracy of wet mass measurements due to constant evaporation.

### Statistical analysis

Based on the activity data for all living animals at the end of the exposure, a generalized linear mixed-effect model (GLMM) for binomial data (moving = 1, not moving = 0) with a logistic model was applied to examine effects of time and salt exposure in the two experiments separately, using the R package *lme4* (Bates et al. 2015; R Core Team 2024). Measured test concentrations and measurement days were used as additive categorical fixed effects and individual animals as random effects to describe the repeated measurements per animal. Coefficients were exponentiated for odds ratios and 95% confidence intervals (95%CI) were provided. Pair-wise comparisons were reported. Models including interactive effects were tested but not supported by the data.

In terms of activity, each organism was marked as active, inactive or dead on each observation day. For each observation day, effective concentrations (ECs) with 95%CI were estimated using counts of active animals among all animals, including active, inactive and dead ones. Different from the usual ECs derived from living animals only, in our case mortality was considered as an extreme negative effect. This way extremely high concentrations which led to mass mortality were included in the concentration-response analysis. The estimated EC10 denoted the concentration that led to 10% negative effect, including activity inhibition and mortality. ECs were derived the same way as the acute LCs according to the ASTM standard (2022), which were estimated using counts of active mussels among all mussels. Our goal was to examine whether this typically acute endpoint can be repeatedly used to serve a chronic purpose.

Concentration-response analysis was performed using the R package *drc* (Ritz et al. 2015). Concentration-response models were selected based on Akaike information criterion (AIC) values and visual inspection of the fitted curves. Based on EC10 values per observation day, a second-step concentration-response model was applied to estimate the time to 50% decrease in EC values (Jensen et al. 2021). Additionally, LC10 from juvenile mortality was derived from the preliminary test. All concentration-response curves were presented in Supplementary Information (Figure S1).

In the definitive experiment, EC10 values were estimated using the endpoint variables soft tissue wet weight and total wet weight (wet soft tissue mass + shell weight) of clams alive after the experiment, separately. Based on other endpoints (length, dry mass and relative water content), no concentration-response relationship was found. Kruskal-Wallis tests were used to compare the mass of the additional animals sampled before the experiments and the control animals after the experiments.

## Results

In the 28-day preliminary test, control survival of *M. balthica* juveniles was 100%. On the last day, *M. balthica* juveniles showed complete activity inhibition in sulphate concentrations ≥ 6000 mg/L, but in the control and 2933 mg/L treatment, activity was observed in all 24 individuals tested. Siphonal autotomy (broken off siphons) was also observed in ≥ 10,000 mg/L during water change. LC10 based on juvenile mortality was 9001 (7893 − 10109) mg/L. EC10 based on juvenile D28 activity was 3980 mg/L, though with a large confidence interval that covers 0, suggesting large uncertainties (Supplementary Information, Table S3).

**Fig. 1:**
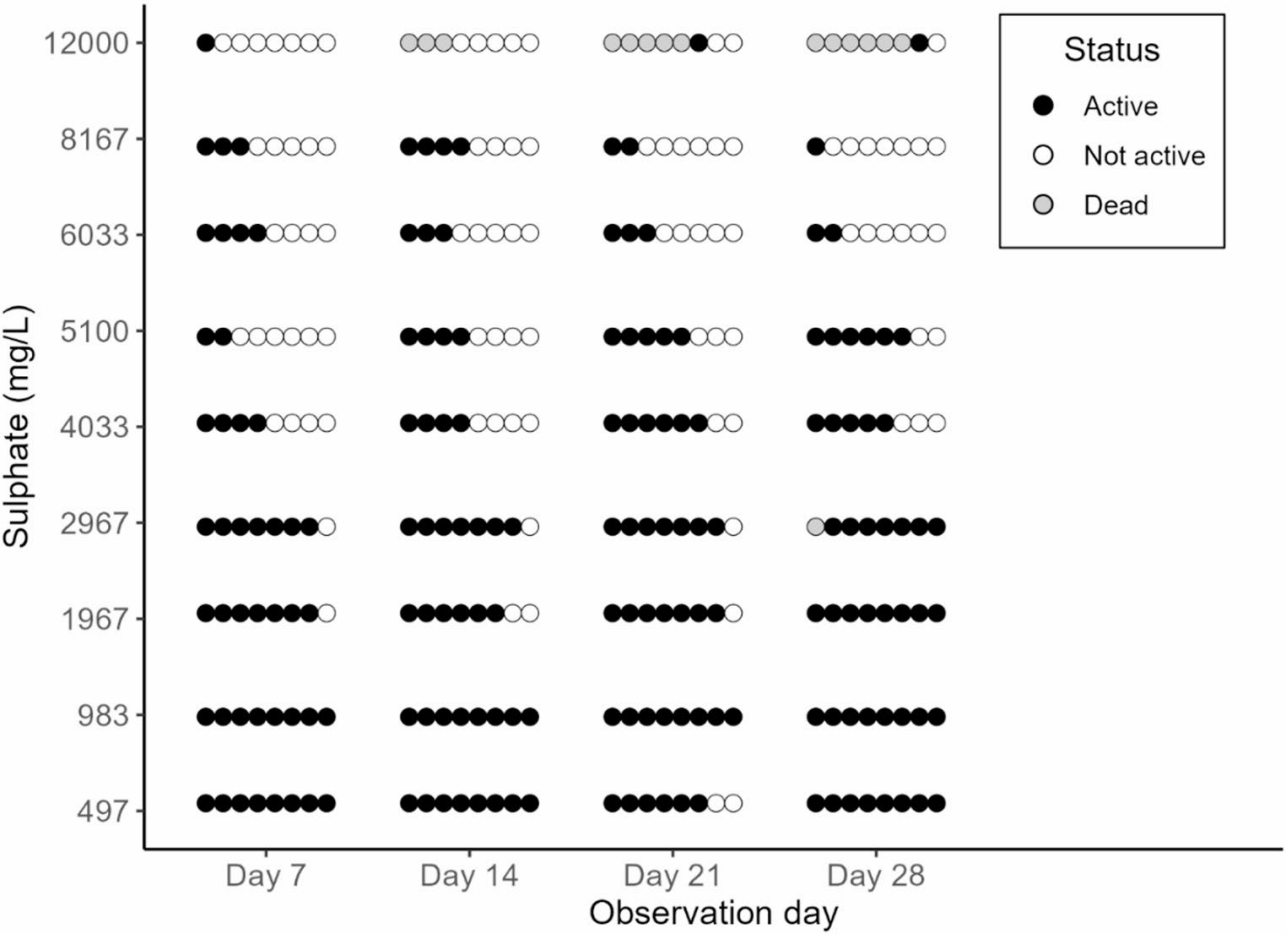
Monitored activity status of *Macoma balthica* adults (*N* = 72, 8 per sulphate concentration) over 28 days. A circle describes one observation of one clam per observation day. Black circles indicate active clams, white circles indicate non-active clams, and grey circles indicate dead clams. Placement order of the circles in each concentration per observation day is not tied to specific individuals

Control survival of *M. balthica* adults in the definitive tests was 100%. Sulphate significantly inhibited *M. balthica* activity (χ^2^ = 36.88, *p* < 0.001, Fig. 1). The odds for observing active movement in 4033 mg/L sulphate is 91% lower (95%CI = 51–98%) than the control (*p* = 0.005, Supplementary Information, Table S4).

**Fig. 2:**
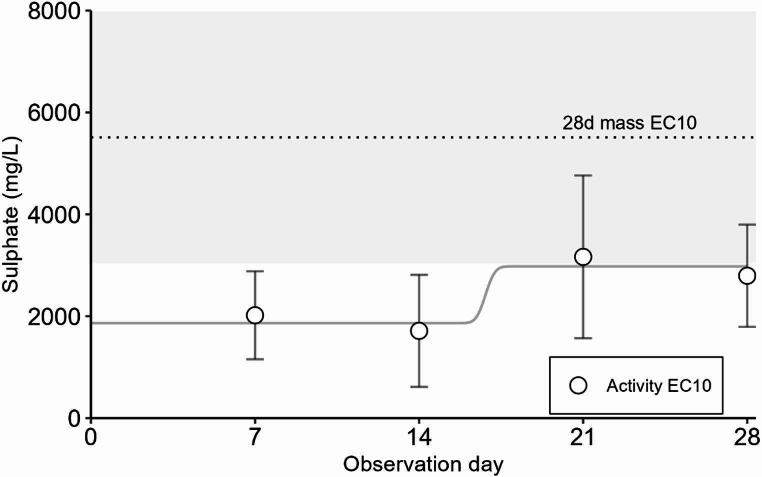
Activity EC10 estimates of *Macoma balthica* across observation days in exposure to sulphate with error bars representing 95% confidence intervals. The curve was fitted to the activity EC10 values per observation day. Dotted line and the light grey area represent 28d wet mass EC10 and 95% confidence interval

Activities in *M. balthica* adults increased slightly with time, but the change was not significant (χ^2^ = 0.71, *p* = 0.87). Adult activity EC10s increased from 2019 mg/L sulphate on Day 7 to 2795 mg/L sulphate on Day 28, suggesting that the activity endpoint became slightly less sensitive over time (Fig. 2, Supplementary Information, Table S3). However, throughout the exposure, adult activity EC10s in *M. balthica* were lower than the 28-day wet mass EC10. In addition, siphonal autotomy was observed in concentrations starting from 4000 mg/L sulphate in 30% of the animals. Based on adult wet soft tissue mass, EC10 value was 5511 mg/L sulphate (Supplementary Information, Table S3).

Compared to the *M. balthica* adults in the control after the experiment (mean = 21.85 ± SE 0.65 mg), no significant changes in dry mass in the extra *M. balthica* adults sampled before the experiment (mean = 23.82 ± SE 0.22 mg, Kruskal-Wallis, *p* = 0.64).

## Discussion

Exposures of elevated sulphate inhibited the activities of *M. balthica*, but the level of activity inhibition decreased (i.e. activity EC10s increased) as the test animals become acclimated to the sulphate exposure. Acclimation was only observed, however, in low and moderate exposure concentrations, as the animals in more extreme concentrations either died or did not recover their activities.

In the sulphate exposure, the activity endpoint in *M. balthica* adults turned out to be a sensitive chronic endpoint compared to adult wet mass and juvenile mortality, and the observation of the deviant autotomy behavior served as additional evidence of support for the sensitivity and relevance of the endpoint. Siphonal autotomy describes the phenomenon when the bivalve sheds parts of its siphon without external force (Wu et al. 2022). Siphonal autotomy in the marine bivalve *Solen grandis* under stress has been observed in aquaculture; loss of siphon segments decreased feeding, digestive activities and metabolism (Wu et al. 2022). Siphonal autotomy occurs through intrinsic musculature, and it can happen without mechanical stimulus (Hodgson 1984). In our experiment, that siphonal autotomy was observed in 30% of individuals exposed to sulphate levels ≥ 4000 mg/L but not at all in those < 4000 mg/L may indicate a sublethal sulphate effect. The exact reason behind the autotomy requires further investigation, but we speculate that such autotomy could be caused by the inability of clams to coordinate movement due to the neurological effects of the exposure, as *M. balthica* siphon musculature is rich in nerve fibers (Pekkarinen 1986). It may also happen as the clams abruptly shut its valves to minimize contact with the test media. The sublethal effect of cropped siphons cannot be reversed and may cause long-term consequences for *M. balthica*, which can suffer from reduced metabolism (Kamermans and Huitema 1994; Peterson and Skilleter 1994; Wu et al. 2022).

Foot or valve activity may function similarly as cardiac activity in bivalves, which can respond to pollutants by increasing activity intensity to counteract the change or by decreasing activity to reduce contact with the contaminants (Hartmann et al. 2016; Vereycken and Aldridge 2023). Bivalves may reduce movement to minimize contact with the highly saline external media (Belamy et al. 2023). Or, in an energetically demanding environment where heightened osmoregulatory costs require compensation from enhanced energy intake, bivalve juveniles may need to engage in intensive pedal feeding and increase energy reserves (Schartum et al.^ 2017^). Hartmann et al. (2016) also found that higher concentrations of NaCl depressed filtration activities in real-time monitoring of adults of freshwater mussels *Anodonta anatina*, while low NaCl concentrations stimulated mussel filtering. Such a hormetic effect may partly affect changes in *M. balthica* activity.

Our study showed that *M. balthica* activity inspected at frequent intervals can be a useful indicator of stress to salinization, albeit with its own challenges. Activity in *M. balthica* was at first sensitive to salt exposure but became less sensitive as the exposure continued. In other words, the sensitivity of the behavioral endpoints depends greatly on the duration of exposure (Bryan et al. 1995). A similar pattern was found in feeding behaviors of chemically stressed fish, which showed initial reduction in food consumption followed by partial or full recovery to the control level (Bryan et al. 1995). However, behavioral acclimation does not necessarily indicate full physiological resistance, as damage may persist in the exposure. This has been observed by Fogliano et al. (2023), who found that in adult marine bivalves *Mytilus galloprovincialis* exposed to delorazepam, valve activity deviations were observed until 7th day and then recovered to the control level, but molecular damage continued until the 21st day. Hu et al. (2024) also found that *Margaritifera* juveniles were capable of active foot movement in test concentrations that significantly reduced growth after 28 days of sulphate exposure. Acclimation in activity can have implications for bivalve behavioral bioindicators in the field; wild bivalves with active valve movement may not always reflect healthy conditions but possible acclimation to chronic stress (Miller 2022; de Bruyn et al. 2024). In addition, behavioral endpoints may not apply to all chemicals, some of which may bypass the avoidance mechanism of bivalves and not affect foot movement in short observation durations (Castro et al. 2018). Variation in experimental temperature and lighting should also be paid attention to when assessing behavioral endpoints, which can affect bivalve movement.

The non-continuous observation method applied in the current study has limitations, such as the potential loss of information at low sampling frequencies (Robson et al. 2009; Vereycken and Aldridge 2023). In addition, sediments were not provided in the test setup, and different activity types could not be observed, such as burrowing inhibition (McGreer 1979; Eldon et al. 1980; Shin et al. 2002). Thus, caution is needed when extrapolating to how *M. balthica* adults in the field would react in exposure to elevated major ions, and our results may be conservative as the test organisms had no other ways to avoid exposure. Additionally, *M. balthica* tested without sediments could potentially suffer from underfeeding.

This is the first study to compare chronic behavioral endpoints to mass-based endpoints in the wild *M. balthica* and adds to the understanding of bivalve responses to salinization. Well-defined chronic water-only exposures as suggested by the ASTM freshwater mussel standard (2022) were chosen. Another strength was our low-cost observation method for capturing timely changes in activity. Given that no previous sulphate biotests on *M. balthica* adults, juveniles or larvae were available, future studies are needed to investigate whether the chronic behaviors of *M. balthica* adults can be more sensitive than those of the larvae stages. 

## Supplementary Information

Below is the link to the electronic supplementary material.

Supplementary Material 1

## Data Availability

Data is provided within the manuscript or supplementary information files.
