# Supplementary material for: Change in activity in Baltic clam (Macoma balthica) exposed to elevated sulphate concentrations
Source: Ecotoxicology. 2025 Oct 14;34(10):2122–30. doi: 10.1007/s10646-025-02977-7 (PMC12619748; doi:10.1007/s10646-025-02977-7)
Supplement: Supplementary file 1 — Supplementary Material 1 [file 10646_2025_2977_MOESM1_ESM.pdf]

Supplementary material

Change in activity in Baltic clam (*Macoma balthica*) exposed to elevated sulphate concentrations

Xiaoxuan Hu*[1], Christian Ritz[2], Hansika Sarathchandra[1], Jouni Taskinen[1] and Juha Karjalainen[1]

[1]University of Jyväskylä, Department of Biological and Environmental Science, Jyväskylä, Finland,

[2]Department of Nutrition, Exercise and Sports, University of Copenhagen, Rolighedsvej 26, DK-1958 Frederiksberg C, Denmark

Corresponding author: Xiaoxuan Hu (email contact: xhux@jyu.fi)

Table S1	Test chemical analysis with nominal levels, measured levels and their standard deviations for sulphate (mg/L) and measured conductivity (μS/cm at 25 °C). Sodium concentrations were calculated.
Table S2	Water chemistry for test water before chemical spiking.
Figure S1	Concentration-response curves across all observation days for all tested *Macoma balthica* adults and on the 28th day for juvenile survival and activity in exposure to sodium sulphate.
Table S3	EC values based on different endpoints for *Macoma balthica*.
Table S4	Statistical results from generalized linear mixed model (GLMM) for adult *Macoma balthica*.

Table S1. Test chemical analysis with nominal levels, measured levels and their standard deviations for sulphate (mg/L) and measured conductivity (µS/cm at 25 °C). Conductivity was measured from the same water samples and a mean value was provided per concentration. Sodium concentrations (mg/L) were calculated as sodium from added sodium sulphate on top of ambient sodium level (for the ambient sodium level 1900 mg/L, see Table S2).

**Macoma**	**Nominal SO4**	**Measured SO4**	**Std SO4**	**Calculated Na**	**Measured conductivity**
Juveniles	500 (control)	513	25	1900 (ambient)	11109
	3000	2933	58	3060	14803
	6000	6000	0	4529	18802
	8000	8033	58	5503	21896
	10000	9833	289	6366	24574
	11500	12000	0	7404	26481
	13000	13000	0	7883	28233
	14500	14667	577	8682	30152
	16000	16000	-	9321	31839
	17500	18000	-	10279	33708
Adults	500 (control)	497	6	1900 (ambient)	11120
	1000	983	21	2133	11957
	2000	1967	58	2604	13480
	3000	2967	58	3083	14937
	4000	4033	115	3594	16463
	5000	5100	200	4106	18063
	6000	6033	115	4553	19387
	8000	8167	153	5575	22330
	12000	12000	0	7412	27043

Table S2. Water chemistry for background test water before chemical spiking.

Site	Tvärminne
Total N, mg/L	0.29
Total P, mg/L	<0.050
DOC, mg/L	4.2
Na, µg/L	1900000
K, µg/L	68200
Mg, µg/L	215000
Ca, µg/L	89900
Cr, µg/L	<1.0
Mn, µg/L	<1

Fe, µg/L	<10.0
Co, µg/L	<0.5
Ni, µg/L	3.6
Cu, µg/L	<5.0
Zn, µg/L	16.3
Al, µg/L	12.1
As, µg/L	<1.0
Se, µg/L	<1.0
Pb, µg/L	<0.5
Sb, µg/L	0.4

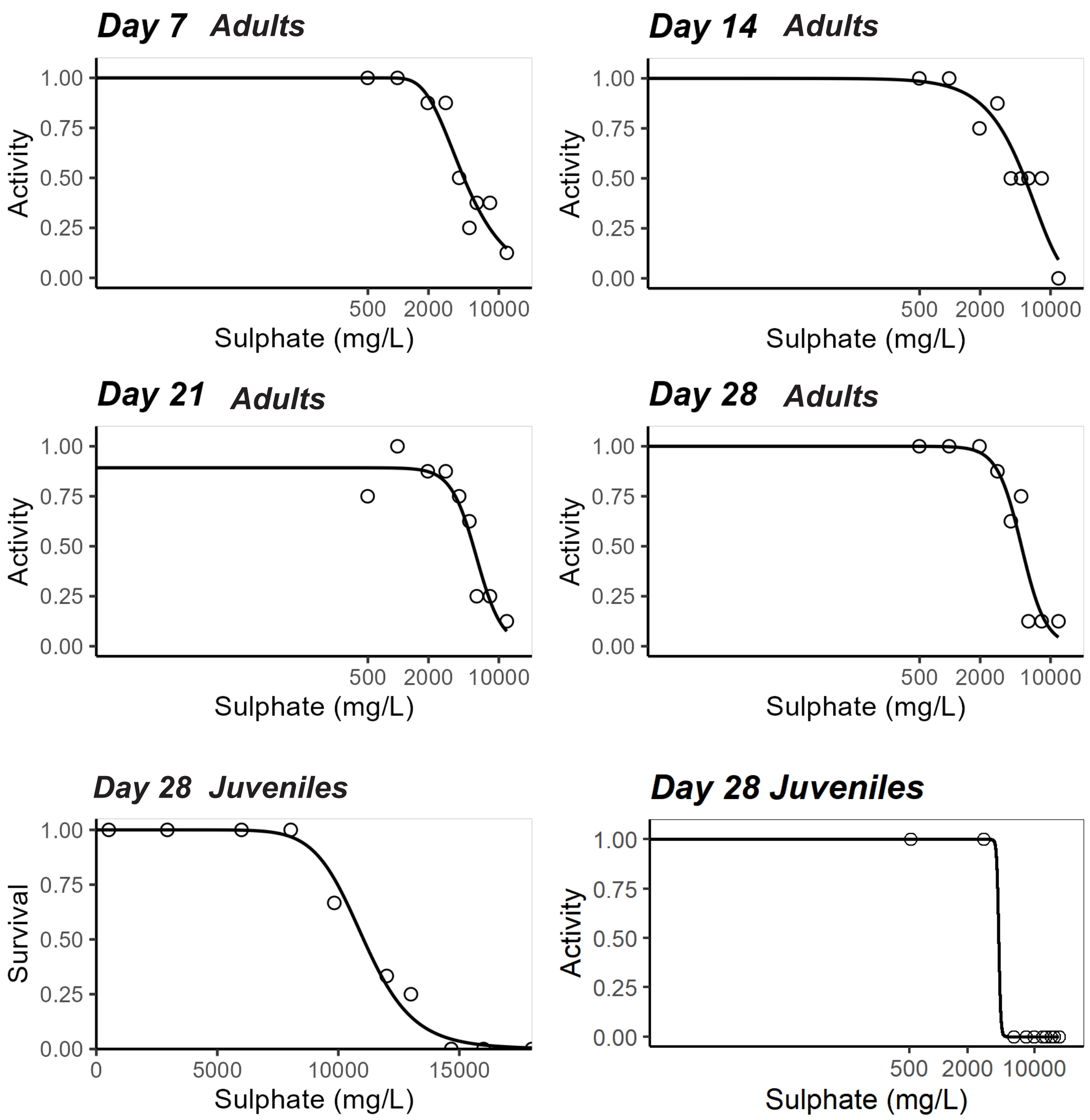

Figure S1. Concentration-response curves across all observation days for all tested *Macoma balthica* adults and on the 28th day for juvenile survival and activity in exposure to sodium sulphate.

Table S3. EC values (mg/L) based on different endpoints for *Macoma balthica*.

Test	Endpoint	EC10	Model link function
M. balthica juveniles	mortality	9001 (7893–10109)	LL.2
	Day 28 activity	3980 (-60637–68598)	LL.2
M. balthica adults	wet soft tissue mass	5511 (3036–7986)	LL.3
	wet total mass	5200 (3165–7236)	LN.4
	Day 7 activity	2019 (1156–2881)	W2.2
	Day 14 activity	1713 (613–2814)	W1.2
	Day 21 activity	3166 (1568–4764)	LL.3
	Day 28 activity	2795 (1792–3799)	LL.2

Table S4. Statistical results from generalized linear mixed model (GLMM) for adult *Macoma balthica*. Measured sulphate concentrations and time were used as categorical fixed effects and individual living mussels as random effects. Interactive effects of treatment and time were not included in the model, as the inclusion would cause huge errors in estimates.

**Fixed effects**	**Estimate**	**Std.error**	**z value**	**p value**	**odds ratio**	**95CI_lower**	**95CI_upper**	**Change in odds**	**95CI_upper**	**95CI_lower**
(Intercept)	2.664	0.809	3.292	0.001						
983 mg/L sulphate	16.819	200.823	0.084	0.933	2.02E+07	0.00	1.77E+178	-2.E+07	100%	-2.E+178
1967 mg/L sulphate	-0.767	0.938	-0.818	0.414	0.46	0.07	2.92	54%	93%	-192%
2967 mg/L sulphate	-0.145	1.066	-0.136	0.892	0.87	0.11	6.99	13%	89%	-599%
4033 mg/L sulphate	-2.396	0.858	-2.794	**0.005**	0.09	0.02	0.49	91%	98%	51%
5100 mg/L sulphate	-2.666	0.859	-3.105	**0.002**	0.07	0.01	0.37	93%	99%	63%
6033 mg/L sulphate	-3.335	0.873	-3.819	< **0.001**	0.04	0.01	0.20	96%	99%	80%
8167 mg/L sulphate	-3.623	0.883	-4.103	< **0.001**	0.03	0.00	0.15	97%	100%	85%
12000 mg/L sulphate	-3.332	1.118	-2.982	**0.003**	0.04	0.00	0.32	96%	100%	68%
Day 14	-3.E-07	0.459	0.000	1.000	1.00	0.41	2.46	0%	59%	-146%
Day 21	0.212	0.461	0.460	0.645	1.24	0.50	3.05	-24%	50%	-205%
Day 28	0.320	0.463	0.691	0.490	1.38	0.56	3.41	-38%	44%	-241%

**Random effects**				
			Variance	S.D.
Individual (intercept)			0.192	0.438
